# Neopterin production and tryptophan degradation during 24-months therapy with interferon beta-1a in multiple sclerosis patients

**DOI:** 10.1186/1479-5876-9-42

**Published:** 2011-04-18

**Authors:** Valentina Durastanti, Alessandra Lugaresi, Placido Bramanti, Mariapia Amato, Paolo Bellantonio, Giovanna De Luca, Orietta Picconi, Roberta Fantozzi, Laura Locatelli, Annalisa Solda', Edoardo Sessa, Rocco Totaro, Silvia Marino, Valentina Zipoli, Marino Zorzon, Enrico Millefiorini

**Affiliations:** 1 Department of Neurological Sciences, University "La Sapienza", Viale dell'Università, 30, 00185, Rome, Italy; 2 Multiple Sclerosis Centre, University "G. d'Annunzio", Chieti, Italy; 3 IRRCS Centro Neurolesi "Bonino-Pulejo", Messina, Italy; 4 Department of Neurology, University of Florence, Florence; 5 Department of Neurology, IRRCS Neuromed, Pozzilli, Italy; 6 Istituto Superiore Sanità (ISS), Rome, Italy; 7 IRRCS Neuromed, Pozzilli, Italy; 8 Department of Clinical Medicine and Neurology, University of Trieste, Trieste, Italy; 9 Department of Molecular Medicine, University La Sapienza, Rome, Italy; 10 Department of Neurology, University of L'Aquila, L'Aquila, Italy; 11 Department of Neurological Sciences, University La Sapienza, Rome, Italy

## Abstract

**Background**

Increased synthesis of neopterin and degradation of tryptophan to kynurenine, measured as kynurenine/tryptophan ratio (kyn/trp ratio), are considered *in vitro *markers of interferon beta-1a (IFNβ-1a) activity. The aim of the study was to investigate the dynamic profile of neopterin and kyn/trp ratio in patients with relapsing remitting multiple sclerosis (RRMS) treated with two different doses of IFNβ-1a over a period of 24 months.

**Methods**

RRMS patients (n = 101) received open-label IFNβ-1a 22 mcg (low dose, LD) or 44 mcg (high dose, HD) subcutaneously (sc), three times weekly for 24 months. Serum measurements of neopterin, kyn/trp ratio and neutralizing antibodies (NAbs) were obtained before treatment (i.e., at baseline) and 48 hours post-injection every 3 months thereafter. Clinical assessments were performed at baseline and every 6 months. Changes in biomarkers over time were compared between LD- and HD-group as well as between patients with/without relapses and with/without NAbs using Analysis of Variance and Mann-Whitney tests.

**Results**

Neopterin (p < 0.001) and kyn/trp ratio (p = 0.0013) values increased over time vs baseline in both treatment groups. Neopterin values were higher (p = 0.046) in the HD-compared to the LD-group at every time point with the exclusion of months 21 and 24 of therapy. Conversely, there were no differences between the two doses groups in the kyn/trp ratio with the exclusion of month 6 of therapy (p < 0.05). Neopterin levels were significantly reduced in NAb-positive patients starting from month 9 of therapy (p < 0.05); the same result was observed for kyn/trp ratio but only at month 9 (p = 0.02). Clinical status did not significantly affect neopterin production and tryptophan degradation.

**Conclusions**

Although differences in serum markers concentration were found following IFNβ administration the clinical relevance of these findings needs to be confirmed with more detailed studies.

## Background

In multiple sclerosis (MS) patients, IFNβ-1a reduces clinical and imaging signs of disease activity, ultimately delaying the progression of physical disability [1,2].

However, a relatively long-term follow-up is necessary for changes in physical disability scores to become evident. Although magnetic resonance imaging (MRI) represents a gold standard for MS diagnosis and can provide fast information regarding the stage of the disease and its changes over time, is still an expensive and time consuming test. Inarguably, a biological marker of drug response would provide a low-cost and easy method of assessing treatment efficacy. To date, no biomarkers that parallel clinical and MRI measurements of response to treatment have been identified. Several lines of evidence suggest that neopterin and tryptophan (trp) degradation catabolites (such as kynurenine [kyn]) could be considered indirect indicators of IFNβ's action [3-5].

Binding of IFNβ to its cell-surface receptor stimulates several immunological processes, including neopterin [D-*erythro*-6-(1',2',3'-trihydroxypropyl)-pterin] production [6] and trp degradation [7,8]. *In vitro *evidence demonstrated that both IFNβ and IFNγ induce neopterin production [9] and activate the enzyme indoleamine (2,3)-dioxygenase (IDO). Such enzyme catalyzes trp degradation to kyn (among other downstream catabolites) in several cell types [10,11]. The kyn/trp ratio provides an estimate of IDO activity and correlates with markers of IFNγ immune activation, like neopterin [8,12].

While neopterin has numerous biochemical and physiological functions in host defense, trp degradation induced by IDO limits trp supply for proliferating cells, thus determining their growth arrest [8,13,14]. Hence, neopterin production and trp degradation could be considered as indicators of the antiviral and immunomodulatory activities of type-I IFNs.

*In vivo *studies in MS patients have confirmed that IFNβ-1a induces neopterin production [15-17] and IDO activation [18]. However, it remains unknown if any of those markers correlates with IFNβ-1a dose and/or clinical outcome.

In this prospective study 101 patients with relapsing remitting MS (RRMS) were treated with one of two doses of IFNβ-1a for 24 months. Repeated evaluations of neopterin and kyn/trp ratio, as well as of physical disability, were performed in order to assess the correlation between biological and clinical effects of IFNβ-1a in these patients. The correlation between the markers of IFNβ biological activity and the presence of neutralizing antibodies (Nabs) [19,20] was also evaluated.

## Methods

### Study design

This open-label randomized study was conducted in seven Italian academic MS clinical centers (University Hospitals of Chieti, Firenze, Isernia, L'Aquila, Messina, Roma, and Trieste), in collaboration with the University of Innsbruck in Austria and the National Institute of Biological Standards and Control in London, UK.

The study consisted of a 12-months screening/enrollment phase, followed by a 24-months follow-up treatment phase (TP), during which IFN-naïve RRMS patients received IFNβ-1a, either 22 mcg (low-dose, LD) or 44 mcg (high-dose, HD) subcutaneously (sc) three times weekly. Given the spontaneous, non-interventional design of the study, in order not to modify common clinical practice, but to warrant at the same time an evenly distributed study population, the dose of IFNβ-1a considered optimal by the treating physician was first started. Patients were then randomized, through a centralized procedure, to be included or not included in the study, maintaining the dosage selected by the treating physician, i.e. a patient was excluded from the study if the selected dosage did not agree with randomization. Care was taken as to reach a balanced sample of LD- and HD-patients (i.e., ~40 to 60% in each group) at each site.

All patients underwent a full clinical examination rating their physical disability, by the Expanded Disability Status Scale, or EDSS score [21], before treatment (referred as baseline thereafter). After the baseline visit, clinical assessments were repeated every 6 months. An additional clinical examination was performed when a clinical relapse occurred, defined as the occurrence of a new symptom or worsening of a pre-existing symptom, lasting at least 48 hours in the absence of fever [22]. Relapses were treated with intravenous methylprednisolone (MP), 1 g/d for 5 days.

At baseline and every 3 months thereafter, blood samples were collected between 8:00am and 1:00 pm, in fasting conditions. The post-dose time was 60-65 hours after the last IFNβ-1a injection. Such interval was chosen based on previous observations that neopterin values remained significantly elevated 48-72 hours after administration of IFNβ-1a both in healthy subjects [23] and in patients with MS [16]. The chosen time interval aimed at both maximizing the timing of sample collection consistency and, at the same time, accommodating patients' availability. As cytokine levels may vary throughout the day, all samples were collected at the same time of the day for each patient.

Blood samples were not collected if clinically evident inflammation/infection was present. In those cases samples were collected 2 weeks after symptom resolution.

### Inclusion and exclusion criteria

Patients with RRMS, according to the Poser's criteria [24], were recruited. Other inclusion criteria were age 18-50 years, body weight within 15% of normal (minimum weight: 50 kg), disease duration ≤ 10 years, at least two relapses in the preceding 2 years, EDSS score of 1.0-5.5. Exclusion criteria were clinical relapse at the time of enrollment; corticosteroid treatment within 1 month, immunomodulatory or immunosuppressive therapy within 6 months prior to study entry, pregnancy, major psychiatric disturbances, and other neurological, neoplastic, autoimmune or major infectious conditions.

### Treatment regimens

Patients received IFNβ-1a at a dose of 44 or 22 mcg, sc three times weekly for 2 years. To minimize adverse effects, IFNβ-1a was titrated as follows: 8.8 mcg at weeks 1 and 2 of therapy, 22 mcg at weeks 3 and 4, and, for patients treated with the higher dose of IFNβ-1a, 44 mcg from week 5.

### Blood sample collection and storage until assay

Blood samples were collected into sterile tubes and allowed to clot spontaneously for 20 minutes at room temperature (i.e., 20-25°C) followed by centrifugation at 3,000 rpm for 10 minutes at 4°C. Sera were immediately aspirated into dry, sterile tubes and stored at -20°C for no longer than 6 months prior to assay. Sera collected for the measurement of neopterin were processed and stored in the dark; sample tubes were covered with aluminum foil throughout the procedure.

### Measurement of neopterin, kyn and trp serum levels

All biological parameters were analyzed by an independent laboratory whose personnel was blinded to patients' clinical and treatment information.

#### • neopterin

Neopterin concentration was measured using a commercially available immunoassay (ELItest, BRAHMS, Berlin, Germany), with a limit of detection of 2 nmol/L. Serum neopterin concentrations in healthy controls were defined as 5.3 ± 2.7 nmol/L, with the upper limit of normal (95th percentile) being 8.7 nmol/L. The assay is a commercial immunoassay which has been reported to be highly reproducible. Coefficients of variation of the assay in our lab are similar to that reported by the manufacturer [i.e. < 5.5% (intra-assay), a < 10.3% (inter-assay)]. The recovery for the neopterin immunoassay was in the range of 91-108%.

#### • kyn and trp

Serum kyn and trp concentrations were measured by high-performance liquid chromatography. Kyn concentrations were monitored by ultraviolet absorption at 350 nm, while trp was measured by detection of natural fluorescence (excitation wavelength: 285 nm, emission wavelength: 350 nm) [25,26] with 3-nitro-L-tyrosine as an internal standard. The coefficient of variation of intra- and interassay determinations for trp and kyn was below 5%. Recovery of trp and kyn was determined by measuring trp and kyn in 20 μl of a pool of 10 sera before and after adding 10 μl of mixture standard solutions of high and low concentration. The recovery for trp and kyn was in the range of 95-105%. Parallel dose-response curves were obtained by serial diluitions of trp and kyn standard solutions and two serially diluted serum samples.

IDO activity was calculated as the ratio of the concentrations of the enzyme product, kyn, divided by its substrate, trp (kyn/trp ratio).

As IDO is not the only enzyme known to trigger the degradation of trp and subsequent kyn production, it was necessary to demonstrate an association between kyn/trp and immune activation using the specific marker, neopterin, in order to confirm IDO involvement.

### Measurement of serum Nabs against IFNβ

Measurement of serum NAbs was carried out by an independent laboratory whose personnel was blinded to patients' clinical and treatment information.

A specific training on blood sampling and serum separation was conducted by the Coordinating Center at their lab facilities. A double blood sampling for each measurement was obtained to ensure a full quality control of the analytical procedures.

To detect the presence of NAbs against IFNβ-1a, serum samples were tested by an *antiviral IFNβ neutralization assay *that assessed the antiviral activity and its neutralization on the basis of the virus-induced cytopathic effect (CPE). Briefly, monolayers of the human glioblastoma cell line 2D9 were pretreated in 96-well microtiter plates with diluted IFNβ-1a (Rebif^®^) preparations (3-10 laboratory units, LU, per ml) that had been pre-incubated for 2 hrs with serial dilutions of the test sera. The cells were then challenged with encephalomyocarditis virus for 24 hrs, stained with 0.05% amido blue black, fixed with 4% formaldehyde in acetic acid buffer and stain was eluted with 0.15 ml of 0.05M NaOH solution before absorbance was read at 620 nm. The NAbs titer was the dilution of serum that reduces 10 LU/ml of IFN to 1 LU/ml (the normal endpoint of antiviral assays). The cut-off for positivity was a titer of 40. Titers were subsequently calculated with the Grossberg-Kawade formula and expressed as ten-fold reducing units (TRU)/ml; cut-off for positivity was 40 TRU/ml [27,28].

NAb-positive patients were defined as those presenting positive titers in at least two consecutive valid measurements.

The NAb assay coefficients of variation (intra-assay and inter-assay) never exceed 0.3 Log.

Recovery of NAb assay was determined by measuring NAb titer in 20 μl of a pool of 20 sera before and after adding anti human IFN-beta antibody reference (G038-501-572, National Institute of Health, Bethesda, USA) at high and low concentrations. The recovery for NAb was in the range of 0.3 Log. Parallel line analysis of bioassay showed no significant difference in slopes of dose response curves prepared by serial diluition of human IFNβ antibody reference (G038-501-572) and three serially diluted NAb positive serum samples.

### Study approvals

The study was carried out according to the Declaration of Helsinki and its updates, ICH-GCP Guidelines for Clinical Trials and EU Directives. All aspects of the study were discussed with the patients, and each patient gave his/her written informed consent prior to enrollment. The local Ethics Committees approved the study protocol.

### Statistical analysis

Data were expressed as means, except for gender that was expressed as percentage (%) and EDSS for which median and standard error (SE) were used.

An Analysis of Variance (ANOVA) for repeated measures was performed to evaluate the effect of time and dose on each of the biological markers. Such an analysis was performed in the entire patient's cohort as well as in sub-groups of patients with or without relapse and patients with or without NAbs.

At each time point, a Mann-Whitney test was performed to identify differences in biological markers and clinical measure between HD and LD groups, between patients with and without clinical relapse and between NAb-positive and NAb-negative patients. Pearson Chi square coefficient was used for comparisons between proportions. Spearman's correlation coefficient was used to evaluate the correlation between laboratory and clinical data.

## Results

### Patient demographics and clinical characteristics

During the 12-months enrollment phase, 101 consecutive IFNβ-1a naïve RRMS patients were enrolled. Patient demographics and clinical characteristics at enrollment are shown in Table 1. There were no differences in baseline demographic and clinical variables between the two doses groups.

**Table 1:** Patient demographics and clinical characteristics at baseline

	IFNβ-1a 44 mcg three times weekly (n = 48)	IFNβ-1a 22 mcg three times weekly (n = 53)
Age (years)	34.2 ± 8.4	35.3 ± 8.2
Sex (fem/male)	30 (62.5%)/18 (37.5%)	41 (77.4%)/12 (22.6%)
Age at disease onset	29.2 ± 8.3	30.4 ± 8.1
Duration of MS (years)	5.2 ± 4.3	4.9 ± 4.2
EDSS score	1.7 ± 1.0	1.6 ± 1.0
Annual relapse rate prior to therapy	0.8 ± 0.9	1.0 ± 1.2

Data are expressed as means, except for sex (expressed in number and percentage); EDSS: Expanded Disability Status Scale; IFNβ-1a: interferon beta 1a; MS: multiple sclerosis. All *p *values for comparisons of the characteristics listed above, between the two treatment groups, were not significant.

Of the 101 patients enrolled, 78 (77.2%) completed the study. No differences in demographic and clinical variables between patients who did and did not complete the study were observed (data not shown). Of the 78 patients who completed the study, 37 (47.4%) experienced at least one relapse. There were no differences in the proportion of relapse-free patients between the two doses groups.

### Influence of dose and duration of therapy on biological markers

**Figure 1:**
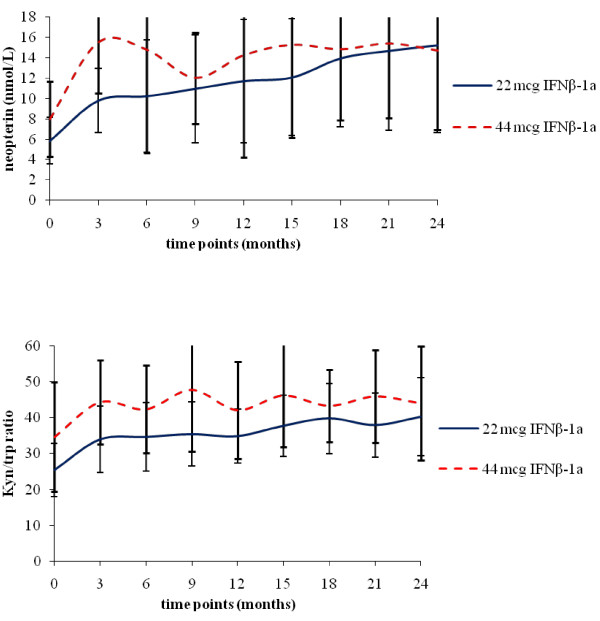
**A: neopterin serum levels as function of time and drug dose; B: kyn/trp ratio as function of time and drug dose**. Neopterin production (A) and tryptophan degradation (as measured by kynurenine/tryptophan ratio) (B) in patients treated with 22 or 44 mcg of interferon beta-1a (IFNβ-1a), administered three times weekly via subcutaneous injection.

Neopterin and kyn/trp ratio profiles of each treatment group are shown in figure 1(A, B). In each treatment group, both neopterin concentration (p < 0.001) and kyn/trp ratio (p = 0.0013) increased over time compared to baseline. Mann-Whitney analyses showed that neopterin values were always higher in the HD-group vs the LD-one at each time point (p = 0.046) apart from months 21 and 24 of treatment period (TP). Conversely, while trends towards higher values of kyn/trp ratio in the HD-group were observed at numerous time points, group differences were not statistically significant at any time point with the exception of month-6 of TP (p < 0.05).

### Correlation between NAb status and neopterin serum level or kyn/trp ratio

At the end of the study, evaluable data on NAbs were available for 71 patients (LD/HD = 35/36). NAbs were present in 15 (21%) patients, 9 of which (26%) in LD-group and 6 (17%) in the HD-group (p = 0.350).

**Figure 2:**
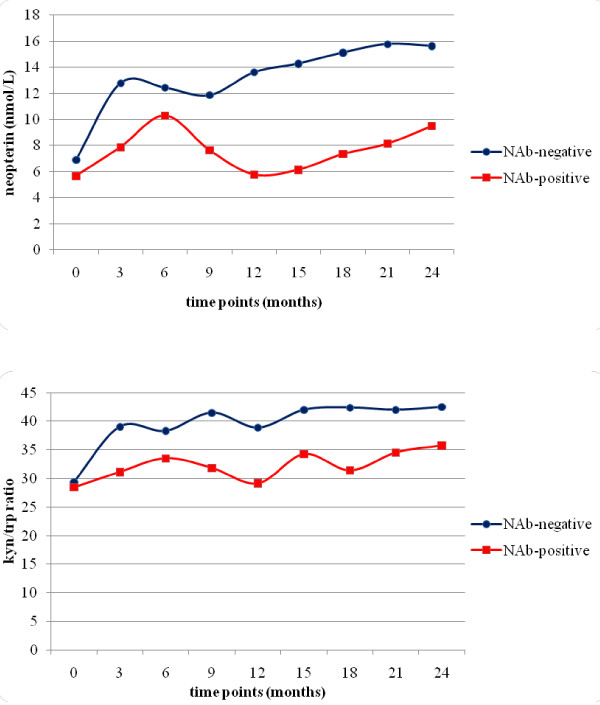
**A: correlation between NAb-status and neopterin serum levels; B: corelation between NAb-status and kyn/trp ratio**. Both, neopterin levels (A) (p = 0.0003) and kyn/trp ratio (B) (p = 0.006) increased over time compared to baseline in each group. Although serum levels of neopterin and kyn/trp ratio showed no statistical difference at baseline between NAb-positive and NAb-negative patients, neopterin levels were significantly reduced in NAb-positive patients starting from month 9 onwards (p < 0.05); the same result was observed for kyn/trp ratio but only relatively to month 9 (p = 0.02).

In figure 2(A, B) neopterin and kyn/trp ratio profiles of NAb-positive and NAb-negative patients are described. In each treatment group, both neopterin levels (p = 0.0003), and kyn/trp ratio (p = 0.006) increased over time compared to baseline. Although serum levels of neopterin and kyn/trp ratio showed no statistically significant difference between NAb-positive and NAb-negative patients at baseline, neopterin levels decreased significantly in NAb-positive patients from month 9 of TP (p < 0.05); the same trend was observed for kyn/trp ratio but the difference was significant only at month 9 of TP (p = 0.02).

### Correlation between biological markers and clinical measures

No significant correlation emerged between laboratory data and disease progression EDSS changes at any of the examined time points; Disease progression was defined as an increase of more than 1 point on the EDSS (for EDSS between 0 and 3.5) and more than 0.5 point (for EDSS >3.5) during the TP. No significant correlation was found between clinical relapses and laboratory data at any of the examined time points. The presence of clinical relapses consisted of the onset of at least one relapse during the TP.

There were no differences in any clinical measures between NAb-positive and NAb-negative patients with the exception of the baseline EDSS which was higher (p = 0.04) in the NAb-positive vs the NAb-negative group (data not shown).

## Discussion

MS is a chronic demyelinating autoimmune disease of the central nervous system (CNS). It is characterized by infiltrates of, mostly, macrophages, T and B lymphocytes, and plasma cells. A variable degree (usually more pronounced in the advanced stages of the disease) of axonal loss and gliotic scars can also be observed. Monocyte-derived macrophages play an important role in these processes and act both as phagocytes and antigen presenting cells (APCs), releasing myelinotoxic factors and proinflammatory cytokines. They are also strongly stimulated by IFNγ secreted by T lymphocytes of the Th1 subset (principal effectors of MS physiopathology).

IFNβ-1a is one of the approved treatments for RRMS patients. The mechanism of actions of IFNβ is still not fully clarified; however, it seems to influence the immune system through an immunomodulatory action and it also enhances the production of several cytokines and proteins [17].

Validated biological markers of the responsiveness to IFNβ-1a treatment would enable a reliable assessment of the efficacy of MS therapy, both in clinical trials and clinical practice, reducing the need for expensive and time-consuming procedures such as MRI. Such markers, though, have not yet been identified [29].

Of the several putative candidates, two appeared to us to be particularly promising: neopterin and kyn/trp ratio. The value of both parameters is significantly raised by the action of Th1-secreted-IFNγ on macrophages similarly to reactive oxygen species (ROS), which can be considered as an index of oxidative stress [30].

Neopterin is a by-product in the synthetic pathway of tetrahydro-biopterin. Upon IFNγ macrophage stimulation, biopterin synthesis is blocked at the step of neopterin whose levels are markedly increased in biological fluids [3,31,32]. Elevated neopterin concentration in body fluids has been observed in a series of conditions characterized by increased Th1 reactivity: infections (particularly HIV), malignancies, autoimmune diseases (particularly RA) and transplants [33,34]. Indeed, it can be considered as an indirect indicator of IFNγ levels (difficult to measure *in vivo*) and of macrophage stimulation intensity. Neopterin has gained high relevance as a marker of immune activation (Th1 cells) to the point that it is used to monitor patients who received allografts for early detection of possible immunological complications.

In addition, another possible biochemical marker has gained wide acceptance: the enhanced tryptophan degradation induced by IFNγ-stimulated macrophages. Namely, the increased cellular expression and activity of IDO and the ensuing raised N-formyl-kynurenine (a by-product in the biochemical pathway to niacin) levels that are measured in the serum. Tryptophan degradation by IDO (measured as kyn/trp ratio) decreases T lymphocytes proliferation and consequently reduces inflammation and allograft rejection. Hence, a new concept is emerging in immunology: cells expressing IDO can inhibit T cells responses and consequently induce tolerance and reduce inflammation. Therefore, kyn/trp ratio could be regarded as a potential index directly related to treatment efficacy.

This study focused on the evaluation of neopterin levels and kyn/trp ratio as markers of IFNβ biological activity. Out of the 101 INF-naïve RRMS patients enrolled in this study, 78 were fully evaluable after 24 months of IFNβ-1a treatment both for the monitored biomarkers and the clinical variables. In this study, we investigated the dynamic profile of neopterin and kyn/trp ratio and its correlation with the clinical features in patients with RRMS treated with two different doses of IFNβ-1a.

Treatment with IFNβ-1a (both LD and HD) increased serum neopterin levels significantly as compared with pre-treatment levels and a dose-response was evident at each time point (p ≤ 0.046). At month 21 of TP and at the end of the study (month 24) a dose-effect was no longer present since neopterin levels were similar in both treatment groups. This might indicate a similar efficacy, although delayed for the LD group, thus exposing patients treated with the LD to the risk of early relapses in the first months of treatment.

The observed patterns of neopterin production over the 2 years of IFNβ-1a treatment probably reflect a biphasic (short- vs long-term effects) aspect of IFNβ-1a biological activity. Initially, IFNβ-1a administration may result in a sharp increase in the neopterin levels owing to the acute, proinflammatory actions of IFNβ-1a [35,36]. However, in the long term its repeated administration may lead to a down-regulation of IFNγ expression and a subsequent decrease in macrophage activation and biomarker expression [9,16]. At each time point, the observed effects of IFNβ-1a on neopterin may reflect the relative predominance of short- over long-term effects or vice versa. The increase in biomarker levels in patients receiving the higher dose of IFNβ-1a became less marked with prolonged treatment, possibly due to tachyphylaxis [19].

A trend showing higher value of kyn/trp ratios in the HD-group was also seen at numerous time points, however, group-differences were not statistically significant at any time point except for month-6 of the TP (p < 0.05). At the end of the study (month 24) a dose-effect was no longer present since kyn/trp ratios were similar in both treatment groups. This finding might indicate that, for tryptophan degradation/IDO activity a ceiling effect might be present at therapeutic dosages.

As previously reported, the increase of kyn/trp ratio in RRMS patients receiving IFNβ-1a indicates the induction of IDO by IFN but such increase does not appear to be dose-dependent [8]. At present, the impact of IFNβ-1a on tryptophan catabolism in patients with RRMS remains unclear.

As with other proteic drugs, some MS patients develop NAbs against IFNβ, which interfere with the receptor-mediated functions of IFNβ; the clinical relevance of NAbs has been the subject of debate because they appear to decrease treatment efficacy of IFNβ in those patients developing persistent, high titer NAbs [37]. It has been reported that myxovirus-resistance protein A (MxA), an antiviral protein exclusively induced by type 1 IFNs, is a sensitive measure of the in vivo response to IFNβ and of its reduced activity due to the development of NAbs [38]. Thus, in the present study, data were also analyzed to determine whether the presence of NAbs affected neopterin serum levels or kyn/trp ratio.

Both, neopterin levels (p = 0.0003) and kyn/trp ratio (p = 0.006) increased over time compared to baseline in each group. Although serum levels of neopterin and kyn/trp ratio at baseline showed no statistical difference between NAb-positive and NAb-negative patients, neopterin levels were significantly reduced in NAb-positive patients starting from month 9 onwards (p < 0.05); the same result was observed for kyn/trp ratio but only at month 9 (p = 0.02). This is a logical consequence of the timing of NAb formation, usually appearing between 3 and 12 months of treatment.

Other studies reported a fall in serum neopterin levels or in the levels of other IFN biologic response markers, including matrix metalloproteinases (MMPs), beta2 microglobulin, MxA, viperin, TNF-related apoptosis-inducing ligand (TRAIL) and X-linked inhibitor apoptosis factor-1 (XAF-1), when NAb titers were elevated in patients with MS [6,20,38-41]. Data clearly support the hypothesis that neopterin is a sensitive measure of biological response to IFNβ and is reduced by the presence of NAbs. Nevertheless, since no relations have been found between neopterin and clinical progression, there are issues regarding the use of neopterin as a measure of the clinical efficacy of IFNβ. It is important to underline that, given the nature of MS, a long-term observation would be needed to clearly demonstrate the effects on disease progression, like for MRI. In the present study, the patients analyzed showed a non-NAb-related abrogation of kyn/trp ratio suggesting that the use of the latter as a biological marker of IFNβ treatment may not be predictive of the biological responsiveness to IFNβ.

To gain further insight into the correlation between biomarkers and clinical efficacy, we also investigated whether disease progression and the occurrence of clinical relapses influenced neopterin production and tryptophan degradation.

We found that the presence of disease progression and clinical relapses did not significantly affect biomarker levels. Furthermore, no differences in dose effect were observed between patients who had a clinical worsening during the study period and those who did not, as previously reported [3,17]. These findings suggest that, although both biomarkers capture the pharmacodynamic effects of IFNβ-1a, they do not necessarily parallel clinical efficacy. A possible explanation is that the immunoinflammatory process in MS takes place in the CNS and disease activity is only partially reflected in the systemic immune compartment; furthermore, many markers are unstable in the periphery and are rapidly eliminated by the kidneys; therefore, the plasma concentration of many putative markers fluctuate significantly and a single measurement could be a mere snapshot. These observations suggest that probably serum is not the ideal body fluid for measuring this marker concentration in order to monitor disease activity in MS. A further possible explanation is that patients with clinical relapses received high dose intravenous corticosteroids and it appears that this form of treatment can suppress the production of neopterin or tryptophan degradation for a period of time. Regarding disease progression, a later explication of the lack of any correlation between disease progression and biomarker levels variation could be that this is a two years study and does not show the entire clinical course of patients.

## Conclusions

Although differences in serum neopterin levels and kyn/trp ratio, following IFNβ administration were found in our study, and a correlation between the presence of NAbs and lower serum levels of neopterin was observed, the clinical relevance of these findings needs to be established with further studies.

This can be ascribed, at least in part to the snapshot effect related to the low-frequency of the sampling interval (3-monthly) of the studied biological markers. Especially in MS, these markers are subject to marked fluctuations, often on a daily basis. In particular for neopterin, a deeper insight of IFNβ treatment influence on its production and its value as a surrogate marker of inflammation in MS, can only be gained/evaluated with a more frequent (at least weekly) sampling. This would only be feasible using urine as a biological specimen, instead of serum. Further studies are warranted to monitor these putative surrogate markers of disease activity in MS more stringently.

## Competing interests

The authors declare that they have no competing interests.

## Authors' contributions

**VD**: collected blood samples, performed clinical examination of the patients and wrote the manuscript. **AL**: collected blood samples, performed clinical examination of the patients and helped to draft the manuscript. **PB**: collected blood samples and performed clinical examination of the patients. **MA**: collected blood samples and performed clinical examination of the patients. **PB**: collected blood samples and performed clinical examination of the patients. **GDL**: collected blood samples and performed clinical examination of the patients. **OP**: performed the statistical analysis. **RF**: collected blood samples and performed clinical examination of the patients. **LL**: collected blood samples and performed clinical examination of the patients. **AS**: helped to draft the manuscript. **ES**: collected blood samples and performed clinical examination of the patients. **RT**: collected blood samples and performed clinical examination of the patients. **SM**: collected blood samples and performed clinical examination of the patients. **VZ**: collected blood samples and performed clinical examination of the patients. **MZ**: collected blood samples and performed clinical examination of the patients. **EM**: designed the manuscript.

All authors read and approved the final manuscript.

## References

[B1] Javed A Reder AT Therapeutic role of beta-interferons in multiple sclerosis Pharmacol Ther 2006 110 35 56 10.1016/j.pharmthera.2005.08.011 16229894

[B2] Kappos L Traboulsee A Constantinescu C Eralinna JP Forrestal F Jongen P Pollard J Sandberg-Wollheim M Sindic C Stubinski B Long-term subcutaneous interferon beta-1a therapy in patients with relapsing-remitting MS Neurology 2006 67 944 953 10.1212/01.wnl.0000237994.95410.ce 17000959

[B3] Giovannoni G Lai M Kidd D Thorpe JW Miller DH Thompson AJ Keir G Feldmann M Thompson EJ Daily urinary neopterin excretion as an immunological marker of disease activity in multiple sclerosis Brain 1997 120 Pt 1 1 13 10.1093/brain/120.1.1 9055793

[B4] Sorensen PS Biological markers in body fluids for activity and progression in multiple sclerosis Mult Scler 1999 5 287 290 10.1191/135245899678846230 10467390

[B5] Bagnato F Durastanti V Finamore L Volante G Millefiorini E Beta-2 microglobulin and neopterin as markers of disease activity in multiple sclerosis Neurol Sci 2003 24 Suppl 5 S301 304 10.1007/s10072-003-0180-5 14652795

[B6] Scagnolari C Duda P Bagnato F De Vito G Alberelli A Lavolpe V Girardi E Durastanti V Trojano M Kappos L Antonelli G Pharmacodynamics of interferon beta in multiple sclerosis patients with or without serum neutralizing antibodies J Neurol 2007 254 597 604 10.1007/s00415-006-0332-7 17420930

[B7] Meyer KC Cornwell R Carlin JM Powers C Irizarry A Byrne GI Borden EC Effects of interferons beta or gamma on neopterin biosynthesis and tryptophan degradation by human alveolar macrophages in vitro: synergy with lipopolysaccharide Am J Respir Cell Mol Biol 1992 6 639 646 1591013 10.1165/ajrcmb/6.6.639

[B8] Amirkhani A Rajda C Arvidsson B Bencsik K Boda K Seres E Markides KE Vecsei L Bergquist J Interferon-beta affects the tryptophan metabolism in multiple sclerosis patients Eur J Neurol 2005 12 625 631 10.1111/j.1468-1331.2005.01041.x 16053472

[B9] Meager T The Molecular Biology of Cytokines 1998 John Wiley & Sons

[B10] Carlin JM Borden EC Sondel PM Byrne GI Interferon-induced indoleamine 2,3-dioxygenase activity in human mononuclear phagocytes J Leukoc Biol 1989 45 29 34 2463322 10.1002/jlb.45.1.29

[B11] Adams O Besken K Oberdorfer C MacKenzie CR Takikawa O Daubener W Role of indoleamine-2,3-dioxygenase in alpha/beta and gamma interferon-mediated antiviral effects against herpes simplex virus infections J Virol 2004 78 2632 2636 10.1128/JVI.78.5.2632-2636.2004 14963171 PMC369218

[B12] Schrocksnadel K Wirleitner B Winkler C Fuchs D Monitoring tryptophan metabolism in chronic immune activation Clin Chim Acta 2006 364 82 90 10.1016/j.cca.2005.06.013 16139256

[B13] Opitz CA Wick W Steinman L Platten M Tryptophan degradation in autoimmune diseases Cell Mol Life Sci 2007 64 2542 2563 10.1007/s00018-007-7140-9 17611712 PMC11136285

[B14] Fuchs D Moller AA Reibnegger G Stockle E Werner ER Wachter H Decreased serum tryptophan in patients with HIV-1 infection correlates with increased serum neopterin and with neurologic/psychiatric symptoms J Acquir Immune Defic Syndr 1990 3 873 876 2166783

[B15] Rudick RA Simonian NA Alam JA Campion M Scaramucci JO Jones W Coats ME Goodkin DE Weinstock-Guttman B Herndon RM Incidence and significance of neutralizing antibodies to interferon beta-1a in multiple sclerosis. Multiple Sclerosis Collaborative Research Group (MSCRG) Neurology 1998 50 1266 1272 9595973 10.1212/wnl.50.5.1266

[B16] Bagnato F Pozzilli C Scagnolari C Bellomi F Pasqualetti P Gasperini C Millefiorini E Galgani S Spadaro M Antonelli G A one-year study on the pharmacodynamic profile of interferon-beta1a in MS Neurology 2002 58 1409 1411 12011292 10.1212/wnl.58.9.1409

[B17] Casoni F Merelli E Bedin R Sola P Bertolotto A Faglioni P Is serum neopterin level a marker of responsiveness to interferon beta-1a therapy in multiple sclerosis? Acta Neurol Scand 2004 109 61 65 10.1046/j.1600-0404.2003.00177.x 14653852

[B18] Matrisciano F Bonaccorso S Ricciardi A Scaccianoce S Panaccione I Wang L Ruberto A Tatarelli R Nicoletti F Girardi P Shelton RC Changes in BDNF serum levels in patients with major depression disorder (MDD) after 6 months treatment with sertraline, escitalopram, or venlafaxine J Psychiatr Res 2009 43 247 254 10.1016/j.jpsychires.2008.03.014 18511076 PMC3744240

[B19] Bertolotto A Gilli F Interferon-beta responders and non-responders. A biological approach Neurol Sci 2008 29 Suppl 2 S216 217 10.1007/s10072-008-0941-2 18690496

[B20] Pachner AR Warth JD Pace A Goelz S Effect of neutralizing antibodies on biomarker responses to interferon beta: the INSIGHT study Neurology 2009 73 1493 1500 10.1212/WNL.0b013e3181bf98db 19884577

[B21] Kurtzke JF Rating neurologic impairment in multiple sclerosis: an expanded disability status scale (EDSS) Neurology 1983 33 1444 1452 6685237 10.1212/wnl.33.11.1444

[B22] Polman CH Reingold SC Edan G Filippi M Hartung HP Kappos L Lublin FD Metz LM McFarland HF O'Connor PW Diagnostic criteria for multiple sclerosis: 2005 revisions to the "McDonald Criteria" Ann Neurol 2005 58 840 846 10.1002/ana.20703 16283615

[B23] Munafo A Trinchard-Lugan II Nguyen TX Buraglio M Comparative pharmacokinetics and pharmacodynamics of recombinant human interferon beta-1a after intramuscular and subcutaneous administration Eur J Neurol 1998 5 187 193 10.1046/j.1468-1331.1998.520187.x 10210831

[B24] Poser CM Paty DW Scheinberg L McDonald WI Davis FA Ebers GC Johnson KP Sibley WA Silberberg DH Tourtellotte WW New diagnostic criteria for multiple sclerosis: guidelines for research protocols Ann Neurol 1983 13 227 231 10.1002/ana.410130302 6847134

[B25] Widner B Werner ER Schennach H Wachter H Fuchs D Simultaneous measurement of serum tryptophan and kynurenine by HPLC Clin Chem 1997 43 2424 2426 9439467

[B26] Laich A Neurauter G Widner B Fuchs D More rapid method for simultaneous measurement of tryptophan and kynurenine by HPLC Clin Chem 2002 48 579 581 11861457

[B27] Grossberg SE Kawade Y Kohase M Yokoyama H Finter N The neutralization of interferons by antibody. I. Quantitative and theoretical analyses of the neutralization reaction in different bioassay systems J Interferon Cytokine Res 2001 21 729 742 10.1089/107999001753124462 11576467

[B28] Grossberg SE Kawade Y Kohase M Klein JP The neutralization of interferons by antibody. II. Neutralizing antibody unitage and its relationship to bioassay sensitivity: the tenfold reduction unit J Interferon Cytokine Res 2001 21 743 755 10.1089/107999001753124471 11576468

[B29] Kieseier BC Hartung HP Bioavailability of interferon-beta in patients with multiple sclerosis - fishing for the surrogate Eur J Neurol 17 344 345 10.1111/j.1468-1331.2009.02893.x 20050905

[B30] Schroecksnadel K Zangerle R Bellmann-Weiler R Garimorth K Weiss G Fuchs D Indoleamine-2, 3-dioxygenase and other interferon-gamma-mediated pathways in patients with human immunodeficiency virus infection Curr Drug Metab 2007 8 225 236 10.2174/138920007780362608 17430111

[B31] Fredrikson S Link H Eneroth P CSF neopterin as marker of disease activity in multiple sclerosis Acta Neurol Scand 1987 75 352 355 10.1111/j.1600-0404.1987.tb05458.x 3618112

[B32] Ott M Demisch L Engelhardt W Fischer PA Interleukin-2, soluble interleukin-2-receptor, neopterin, L-tryptophan and beta 2-microglobulin levels in CSF and serum of patients with relapsing-remitting or chronic-progressive multiple sclerosis J Neurol 1993 241 108 114 10.1007/BF00869773 8138825

[B33] Sucher R Schroecksnadel K Weiss G Margreiter R Fuchs D Brandacher G Neopterin, a prognostic marker in human malignancies Cancer Lett 287 13 22 10.1016/j.canlet.2009.05.008 19500901

[B34] Murr C Widner B Wirleitner B Fuchs D Neopterin as a marker for immune system activation Curr Drug Metab 2002 3 175 187 10.2174/1389200024605082 12003349

[B35] Wandinger KP Sturzebecher CS Bielekova B Detore G Rosenwald A Staudt LM McFarland HF Martin R Complex immunomodulatory effects of interferon-beta in multiple sclerosis include the upregulation of T helper 1-associated marker genes Ann Neurol 2001 50 349 357 10.1002/ana.1096 11558791

[B36] Boylan MT Crockard AD Duddy ME Armstrong MA McMillan SA Hawkins SA Interferon-beta1a administration results in a transient increase of serum amyloid A protein and C-reactive protein: comparison with other markers of inflammation Immunol Lett 2001 75 191 197 10.1016/S0165-2478(00)00310-2 11166375

[B37] Polman C Kappos L White R Dahlke F Beckmann K Pozzilli C Thompson A Petkau J Miller D Neutralizing antibodies during treatment of secondary progressive MS with interferon beta-1b Neurology 2003 60 37 43 12525715 10.1212/wnl.60.1.37

[B38] Malucchi S Gilli F Caldano M Marnetto F Valentino P Granieri L Sala A Capobianco M Bertolotto A Predictive markers for response to interferon therapy in patients with multiple sclerosis Neurology 2008 70 1119 1127 10.1212/01.wnl.0000304040.29080.7b 18272865

[B39] Cook SD Quinless JR Jotkowitz A Beaton P Serum IFN neutralizing antibodies and neopterin levels in a cross-section of MS patients Neurology 2001 57 1080 1084 11571337 10.1212/wnl.57.6.1080

[B40] Gilli F Bertolotto A Sala A Hoffmann F Capobianco M Malucchi S Glass T Kappos L Lindberg RL Leppert D Neutralizing antibodies against IFN-beta in multiple sclerosis: antagonization of IFN-beta mediated suppression of MMPs Brain 2004 127 259 268 10.1093/brain/awh028 14607790

[B41] Gilli F Marnetto F Caldano M Sala A Malucchi S Capobianco M Bertolotto A Biological markers of interferon-beta therapy: comparison among interferon-stimulated genes MxA, TRAIL and XAF-1 Mult Scler 2006 12 47 57 10.1191/135248506ms1245oa 16459719

